# Tumor-suppressive MEG3 induces microRNA-493-5p expression to reduce arabinocytosine chemoresistance of acute myeloid leukemia cells by downregulating the METTL3/MYC axis

**DOI:** 10.1186/s12967-022-03456-x

**Published:** 2022-06-27

**Authors:** Airong Wang, Yufei Chen, Luyao Shi, Mengya Li, Lingling Li, Shujuan Wang, Chong Wang

**Affiliations:** Department of Hematology, the First Affiliated Hospital of Zhengzhou University, Erqi District, No. 1, Eastern Jianshe Road, Zhengzhou, 450052 Henan People’s Republic of China

**Keywords:** Acute myeloid leukemia, Chemotherapy, Maternally expressed gene 3, microRNA-493-5p, Methyltransferase-like 3

## Abstract

**Background:**

Chemoresistance serves as a huge obstacle for acute myeloid leukemia (AML) patients. To counteract the chemoresistance in AML cells, we discussed the role of maternally expressed gene 3 (MEG3) in arabinocytosine (AraC) chemoresistance in AML cells.

**Methods:**

MEG3, microRNA (miR)-493-5p, methyltransferase-like 3 (METTL3) and MYC expression in AML cells was determined and then their interactions were also analyzed. Then, the viability and apoptosis of AML cells were determined through loss- and gain- function assay. The level of m6A modification in AML cells was examined. AML mouse models were also established to validate the potential roles of MEG3.

**Results:**

MEG3 and miR-493-5p were downregulated in AML cells, and they were lower in resistant cells than in parental cells. MEG3 led to elevated expression of miR-493-5p which targeted METTL3. METTL3 increased expression of MYC by promoting its m6A levels. Overexpression of MEG3 and miR-493-5p or knockdown of METTL3 inhibited HL-60 and Molm13 cell proliferation and promoted their apoptosis. Overexpressed MEG3 induced heightened sensitivity of AML cells to AraC. However, the suppression of miR-493-5p reversed the effects of overexpressed MEG3 on AML cells.

**Conclusions:**

Collectively, MEG3 could upregulate miR-493-5p expression and suppress the METTL3/MYC axis through MYC m6A methylation, by which MEG3 promoted the chemosensitivity of AML cells.

**Supplementary Information:**

The online version contains supplementary material available at 10.1186/s12967-022-03456-x.

## Introduction

Acute myeloid leukemia (AML) is a relatively aggressive myeloid malignancy, typically accompanied by strikingly heterogenous outcomes [1]. AML is characterized by abnormal proliferation of myeloid cells [2] and accounts for high morbidity and mortality particularly among older AML patients, with poor prognosis [3]. While the large majority of AML patients respond to chemotherapy, drug resistance remains a persistent problem and contributes to rising mortality [4]. For example, arabinocytosine (AraC) serves as the backbone for AML therapy [5]. However, treatment failure underlies approximately 50% of relapse incidence in AML [6]. Therefore, understanding the mechanism of reducing the chemoresistance could help exert the antileukemic effects of AraC in AML regimens.

LncRNA maternally expressed gene 3 (MEG3) has a length of ~ 1.6 kb [7], and downregulation of its expression may be associated with poor prognosis of AML patients [8]. Moreover, MEG3 methylation has been shown as a potential biomarker of leukemia [9], implying a putative high pathophysiological relevance in similar malignant conditions. Whether MEG3 has a role in mediating the chemotherapy efficacy in AML has not been researched. Notably, microRNA-lncRNA interactions are widely documented [10]. The potential roles of miRNAs in AML pathogenesis have been documented in considerable analysis [11]. Notably, aberrantly expressed miR-493-5p has been found correlated with hypermethylation of MEG3 in liver cancer cells and tissues [12]. Altered expression of miR-493-5p has been observed in chronic myeloid leukemia cells [13]. Gain-function assay of miR-493-3p could elevate the N6-methyladenosine (m6A) levels to downregulate YTH N6-methyladenosine RNA binding protein 2 in prostate cancer [14].

Methyltransferase-like 3 (METTL3) serves as a main constituent of the m6A methyltransferase complex [15]. Moreover, we found from the dataset analysis that METTL3 was enriched in T cell differentiation and methyltransferase complex and highly expressed in myeloid leukemia. METTL3 can mediate the myeloid differentiation of leukemia cells [16]. Therefore, we speculated that MEG3/miR-493-5p/METTL3 might act as a network that affects the pathogenesis of AML in regulation of the anti-leukemic effect of AraC. Therefore, we aimed to explore their effects and identified their interaction as these investigations may enable the development of novel chemotherapy regimens for AML.

## Materials and methods

### Ethics

This study was ratified by the Ethics Committee of the First Affiliated Hospital of Zhengzhou University. All participants or their guardians provided written informed consents prior to enrollment. Animal experiments were conducted in line with the *Guide for the Care and Use of Laboratory Animals.*

### AML-related dataset acquisition and differential gene analysis

AML-related miRNA expression dataset GSE62137 was retrieved from the Gene Expression Omnibus database, including 3 normal samples (control) and 15 AML samples. Differential analysis was conducted using the R “limma” package (version 3.4.1) to screen the differentially expressed miRNAs with |log2FC|> 0.5 and adjusted *p* < 0.05 (P_FDR_ < 0.05) as the threshold after multiple testing by the Benjamini and Hochberg method.

Differentially expressed genes (DEGs) in normal samples and AML samples from TCGA and GTEx databases were analyzed using “Differential Expression Analysis” module. miR-493-5p downstream genes were predicted using the mirDIP and TargetScan databases. Correlation analysis between miRNA and MEG3 in the AML samples in the TCGA database was performed using LinkedOmics.

DEGs were then subjected to gene ontology and Kyoto Encyclopedia of Genes and Genomes enrichment analysis using the “clusterprofiler” package (version 3.14.3) in R software, followed by visualization of results with the “ggplot2” package (version 1.26.0). A *p* < 0.05 described statistically significant.

### Collection of bone marrow samples

Bone marrow samples were extracted from 35 AML patients (20 males and 15 females). The enrolled patients were confirmed with AML in the light of the classification criteria of French, American, Britain (FAB), the World Health Organization (WHO), and the immunophenotypic and cytogenetic analysis. Patients conforming to the following criteria were enrolled in this study: (1) Patients diagnosed by FAB diagnostic criteria for AML; (2) Patients diagnosed by WHO diagnostic criteria; (3) Patients without autoimmune diseases; (4) Patients without historical records of exposure to toxic substances; (5) Patients without history of radiation or other malignancy; and (6) Patients without historical records of anti-leukemic therapy. Bone marrow samples from 35 health volunteers (24 males and 11 females; aged from 14–72 years, the bone marrow donors of hematopoietic stem cells) were served as the control. The detailed information is shown in Additional file 1: Table S1. Bone marrow samples were stored at − 80℃ before RNA purification.

### Cell culture

Human AML cell lines (HL-60 and Molm13) and normal bone marrow cell line HS-5 (Meisen CTCC, Zhejiang, China) were cultured in Roswell Park Memorial Institute (RPMI)-1640 medium (GIBCO, California) replenished with 10% fetal bovine serum (FBS) at 37 °C in a 5% CO_2_ incubator.

Construction of AraC (PHR1787, Sigma) resistant cell line: AML cell lines were cultured under a gradual increasing dose of AraC (initial dose of 25 nM) for one year (AraC dose increased by about 6–10 times) to produce AML cell lines stably resistant to AraC (HL-60 R and Molm13 R). The successfully constructed drug-resistant cell lines were cultured in RPMI-1640 complete medium under a humid environment containing 5% CO_2_ at 37 °C.

### Plasmid transfection and drug treatment

HL-60, Molm13, HL-60 R, and Molm13 R cells were seeded in a 6-well plate at a density of 3 × 10^5^ cells/well. When reaching 50% confluence, the cells were transfected according to Endo-Porter delivery reagent from Gene Tools Inc (Philomath, OR). AraC was dissolved in PBS to the required concentration for culturing cells. The culture medium was changed after 6 h, and the cells were collected 48 h after transfection.

HL-60 and Molm13 cells were transfected with short hairpin RNA targeting negative control (sh-NC), sh-MEG3-1, sh-MEG3-2, inhibitor NC, miR-493-5p inhibitor, overexpression (oe)-NC, oe-METTL3, oe-MYC, AraC, mimic NC, miR-493-5p mimic, sh-METTL3, or sh-MYC.

HL-60 R and Molm13 R cells were treated with oe-NC, oe-MEG3, mimic NC, miR-493-5p mimic, sh-NC, sh-METTL3-1, sh-METTL3-2, sh-MYC-1, sh-MYC-2, AraC, oe-NC, inhibitor NC, miR-493-5p inhibitor, oe-METTL3, or oe-MYC.

The required plasmids or sequences were chemically synthesized by Shanghai GenePharma Co. Ltd. (Shanghai, China). The cells were collected after transfection for 48 h, then pre-incubated with AraC for 2 h, and finally used for subsequent experimentations. The silencing sequences are listed in Additional file 2: Table S2.

### Lentivirus transduction

Molm13 and Molm13 R cells stably expressing luciferase (luc) and green fluorescent protein (GFP) were constructed. Firefly luciferase sequence after codon optimization was separated from pGL4.51 (E1320; Promega, Madison, WI) and cloned into the lentiviral vector pCDH-CMV-MCS-EFS-copGFP to produce pCDH-luc-CopGFP (#72,485; Addgene, Cambridge, MA), while pMIF-cGFP-Zeo-MEG3 (System Biosciences, Irvine, CA) was sub-cloned into the pGIPZ vector. Lentivirus was transduced into Molm13 and Molm13 R cells at a multiplicity of infection of 20. Then, 1 μg/mL puromycin was applied for 3 days to establish stable cells [17].

### Animal experiments

A total of 64 NSG mice (Beijing Vital River Laboratory Animal Technology Co., Ltd., Beijing, China) were housed in SPF animal laboratory individually with humidity of 60–65% at 22–25℃ under a 12 h light/dark cycle, free access to food and water. The mice were acclimated for one week.

Mice were injected with Molm13-luc-GFP AML cells treated with sh-NC, sh-MEG3, sh-NC + AraC, sh-MEG3 + AraC, oe-NC, oe-MEG3, oe-NC + AraC, or oe-MEG3 + AraC, respectively (n = 8 in each group).

NSG mice were intravenously injected with cells treated by Molm13-luc-GFP or Molm13 R-luc-GFP (2 × 10^6^ cells/100 μL). After injection of the luciferase substrate colenterazine (BIOTIUM, CA), mice were subjected to anesthetization and non-invasively imaging using an in vivo imaging system (IVIS-200; Xenogen Inc., Alameda). Whole-body bioluminescence was quantified in the selected regions. After injection of AML cells for 7 days, mice were administered with AraC (100 mg/kg body weight) twice a week. The control mice were injected with PBS. At day 18, mice injected with AML cells were euthanized using CO_2_ asphyxiation.

### Reverse transcription quantitative polymerase chain reaction (RT-qPCR)

Total RNA was extracted using TRIzol reagent (15596018, Invitrogen). miRNA and mRNA were reversely transcribed into cDNA with TaqMan™ MicroRNA RT Kit (4366596, Thermo Fisher Scientific) and High-Capacity cDNA RT Kit (4368813, Thermo Fisher Scientific), respectively. RT-qPCR was conducted using an ABI7500 quantitative PCR instrument (Thermo Fisher Scientific) with SYBR Premix Ex Taq™ (Tli RNaseH Plus) kit (RR820A, TaKaRa, Japan), where beta-actin (β-actin) and U6 were used as normalizers. The relative mRNA expression was determined using the 2^−ΔΔCt^ method. The primer sequences are shown in Additional file 3: Table S3.

### Immunoblotting

Immunoblotting was performed with diluted antibodies (Abcam Inc., Cambridge, UK) against Bcl-2 (1: 1000, ab196495), Bax (1: 1000, ab32503), cleaved caspase-3 (1: 500, ab32042), and β-actin (1: 5000, ab8227) as well as goat anti-rabbit IgG antibody (ab97051, 1: 2000, Abcam) [18].

### Immunohistochemistry (IHC)

IHC was carried out with the help of the primary antibodies against MYC (1: 100, ab32072, Abcam) and METTL3 (1: 500, ab195352, Abcam). Finally, observation of sections was implemented under an inverted microscope (CX41, Olympus). Each sample was photographed randomly with 5 different visual fields. Dark brown indicated the target cell infiltration labeled by antibody. The target infiltration degree of each picture was analyzed by Image J software to draw the statistical result map.

### Dual-luciferase reporter assay

The constructed METTL3 3’untranslated region (3’UTR) wild type (WT) and mutant type (MUT) (CUGAAGAGUGAUAUUACAUGUAU) gene fragments were introduced into pMIR-reporter (Huayueyang Biotechnology, Beijing, China) and co-transfected with human miR-493-5p mimic or NC-mimic into HEK293T cells (BN Biotech, Shanghai, China), respectively. After 48 h, the cells were lysed followed by the detection of the luciferase signal with Dual-Luciferase Reporter Assay System (Promega) and a Glomax20/20 luminometer (Promega).

### Dot blot analysis

Total RNA extraction was performed with the help of the Trizol method followed by purification through PolyATtract mRNA Isolation Systems (No. A-Z5300, AD Technology, Beijing, China). Then, mRNAs were subjected to denaturation for 7 min under ultraviolet irradiation and placed on an Amersham Hybond-N^+^ membrane (GE Healthcare, Waukesha, WI) optimized based on nucleic acid transfer. After UV cross-linking, the membrane was subjected seal with 5% skim milk powder, and incubation with anti-m6A antibody (1: 5000, ab284130, Abcam) at 4 °C, followed by visualization using Immobilon Western Chemilum HRP Substrate (Merck Millipore, Darmstadt, Germany).

### Me-RIP-qPCR

RIP assay was implemented with anti-m6A antibody (1: 500, ab151230, Abcam) and IgG antibody (ab109489, 1: 100, Abcam). RT-qPCR detection was carried out using Qiagen’s RT kit and × SYBR green qPCR Master Mix. Cycle threshold value was adopted to calculate the mRNA enrichment. The sequences for MYC-m6A were: Forward: 5′-GCATACATCCTGTCCGTCCA-3'; Reverse: 5′-TGAGCGAAAAAGAGGTTGCTG-3′.

### Flow cytometry

Cell apoptosis measurement was performed utilizing FITC Annexin V Apoptosis Detection Kit I (556547, BD Biosciences, Franklin Lakes, NJ) through a flow cytometer (FACSVerse/Calibur/AriaIISORP, BD) [19].

### Cell counting kit-8 (CCK-8) assay

Cells under different treatments were seeded into 96-well plates (1 × 10^3^ cells/well) in 100 μL medium containing 10% FBS. The number of cells was measured using a CCK-8 kit (CK04, Dojindo, Kumamoto, Japan) with absorbance at 450 nm with a microplate reader.

### Statistical analysis

SPSS 21.0 software (IBM corporation, Armonk, NY) was applied for data analysis with the obtained data (from three independent repeated experiments) shown as mean ± standard deviation. Two group comparisons were studied by independent sample *t* test. Multiple group comparisons were carried out with one-way analysis of variance (ANOVA) and Tukey’s post-hoc test or repeated measures ANOVA and Bonferroni’s post hoc test. Correlation was studied using Pearson’s correlation coefficient. *p* < 0.05 manifested statistical significance.

## Results

### Overexpression of MEG3 induces apoptosis and promotes the sensitivity of AML cells to AraC

**Fig. 1:**
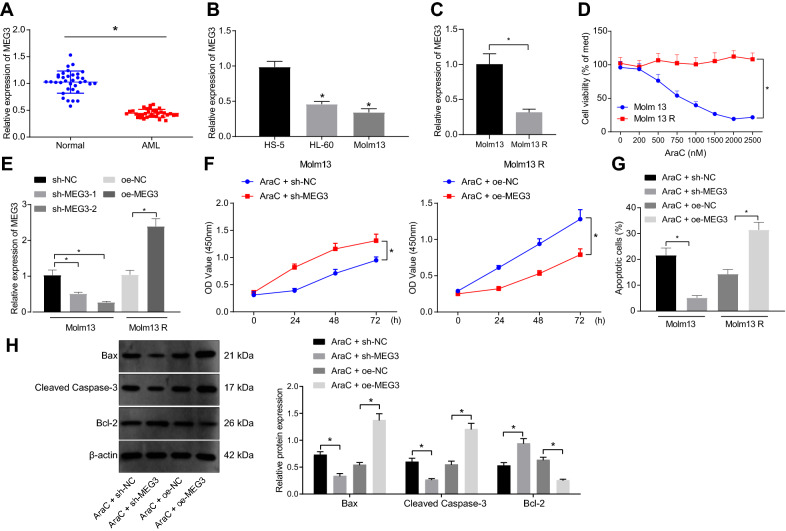
Restored MEG3 stimulates the apoptosis of Molm13 cells and increases the sensitivity of Molm13 cells to AraC. A, MEG3 expression in bone marrow samples of AML patients (n = 35) and healthy individuals (n = 35) determined by RT-qPCR. * *p* < 0.05 compared with healthy individuals. B, MEG3 expression in normal bone marrow cells HS-5 and AML cell lines HL-60 and Molm13 determined by RT-qPCR. * *p* < 0.05 compared with HS-5 cells. C, MEG3 expression in Molm13 and Molm13 R cells following treatment with AraC at different concentrations determined by RT-qPCR. D, Molm13 and Molm13 R cell viability following treatment with AraC at different concentrations assessed by CCK-8 assay (0 μM AraC was used as the control). E, MEG3 expression in Molm13 cells treated with sh-MEG3-1 and sh-MEG3-2 and Molm13 R cells treated with oe-MEG3-1 determined by RT-qPCR. F, CCK-8 assay was used to detect the viability of Molm13 cells co-treated with 2 μM AraC and sh-MEG3 and Molm13 R cells co-treated with 2 μM AraC and oe-MEG3. G, Flow cytometry was used to detect the apoptosis of Molm13 cells co-treated with 2 μM AraC and sh-MEG3 and Molm13 R cells co-treated with 2 μM AraC and oe-MEG3. H, Western blot analysis was used to measure the expression of Bcl-2, Bax and cleaved caspase-3 proteins in Molm13 cells co-treated with 2 μM AraC and sh-MEG3 and Molm13 R cells co-treated with 2 μM AraC and oe-MEG3. * *p* < 0.05. Cell experiments were performed three times independently

MEG3 is capable of inhibiting the occurrence of AML [20]. Therefore, we detected MEG3 expression in the collected bone marrow samples and AML cells by RT-qPCR, with the results showing that MEG3 expression was reduced in AML bone marrow samples (Fig. 1A) as well as in HL-60 and Molm13 cells (Fig. 1B).

Subsequently, we attempted to investigate whether MEG3 expression was correlated with AML cell growth and chemoresistance. AraC resistant AML cell lines (HL-60 R and Molm13 R) were constructed and treated with different concentrations of AraC. RT-qPCR (Fig. 1C and Additional file 6: Fig. S1A) showed that compared with parental cells (HL-60 and Molm13), MEG3 expression was lower in drug-resistant cell lines. Furthermore, CCK-8 assay (Fig. 1D and Additional file 6: Fig. S1B) presented that the activity of parental cells decreased significantly with the increase of dose, and there was no significant dose-dependent decrease in the activity of drug-resistant cells, indicating that there was AraC resistance in drug-resistant cells. Moreover, when the dose of AraC was 2 μM, the activity of parental cells was the lowest. Therefore, AraC (2 μM) was selected for subsequent experimentations. Next, MEG3 was silenced or overexpressed in AML parents and drug-resistant cell lines, respectively. RT-qPCR (Fig. 1E and Additional file 6: Fig. S1C) exhibited that MEG3 expression in HL-60 and Molm13 decreased significantly, and the silencing efficiency of sh-MEG3-2 sequence was the highest. Thus, sh-MEG3-2 was selected for subsequent experimentations. Overexpression of MEG3 in HL-60 R and molm13 R cells significantly elevated MEG3 expression.

Besides, we confirmed that the apoptosis of HL-60 and Molm13 cells treated with AraC + sh-MEG3 was prominently inhibited, whereas cell viability was promoted, and opposing tendency was witnessed upon AraC + oe-MEG3 treatment (Fig. 1F, Gand Additional file 6: Fig. S1D, E), indicating that MEG3 affected the sensitivity of AML cells to AraC. Western blot analysis results (Fig. 1H and Additional file 6: Fig. S1F) presented that silencing of MEG3 in parental cells inhibited the expression of Bax and cleaved caspase-3 and promoted Bcl-2 expression, while overexpression of MEG3 in drug-resistant cells led to opposite results, suggesting that overexpression of MEG3 enhanced the sensitivity of AML cells to AraC.

These results indicated that MEG3 was poorly expressed in AML bone marrow tissues and cell lines, and overexpression of MEG3 promoted the sensitivity of AML cells to AraC.

### MEG3 enhances the sensitivity of AML cells to AraC by upregulating miR-493-5p expression

**Fig. 2:**
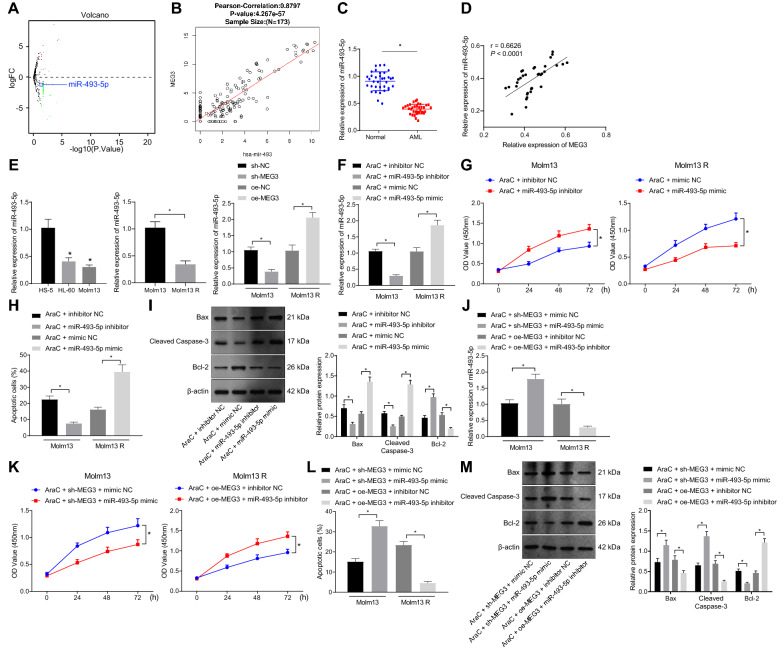
MEG3 upregulates miR-493-5p to strengthen the sensitivity of Molm13 cells to AraC. A, A volcano map of differentially expressed miRNAs in AML-related miRNA expression dataset GSE62137 (3 control samples and 15 tumor samples). Red represents significantly upregulated genes, and green represents significantly downregulated genes. B, Correlation analysis between miR-493 expression and MEG3 expression in AML samples included in TCGA. The top represents Pearson’s correlation coefficient and correlation *p* value. C, miR-493-5p expression in bone marrow samples of AML patients (n = 35) and healthy individuals (n = 35) determined by RT-qPCR. D, Pearson correlation analysis of MEG3 expression and miR-493-5p expression in bone marrow samples of AML patients (n = 35). E, miR-493-5p expression in HS-5, HL-60, Molm13, HL-60 R, and Molm13 R cells measured by RT-qPCR. Molm13 cells were treated with AraC (2 μM) + miR-493-5p inhibitor and Molm13 R cells were treated with AraC (2 μM) + miR-493-5p mimic. F, miR-493-5p expression in Molm13 and Molm13 R cells determined using RT-qPCR. G, CCK-8 was used to detect the viability of Molm13 and Molm13 R cells. H, Flow cytometry was used to detect apoptosis of Molm13 and Molm13 R cells. I, Expression of Bcl-2, Bax, and cleaved caspase-3 proteins in Molm13 and Molm13 R cells determined using Western blot analysis. Molm13 cells were treated with AraC (2 μM) + sh-MEG3 + miR-493-5p mimic and Molm13 R cells were treated with AraC (2 μM) + oe-MEG3 + miR-493-5p inhibitor. J, miR-493-5p expression in Molm13 and Molm13 R cells determined by RT-qPCR. K, CCK-8 assay was used to measure the viability of Molm13 and Molm13 R cells. L, Flow cytometry of apoptosis of Molm13 and Molm13 R cells. M, Bcl-2, Bax, and cleaved caspase-3 protein expression in Molm13 and Molm13 R cells determined by Western blot analysis. * *p* < 0.05. Cell experiments were performed three times independently

We then moved to examine how MEG3 enhances the sensitivity of AML cells to AraC. Differential analysis of the GSE62137 dataset documented that miR-493* (miR-493-5p) expression was aberrantly low in AML samples (logFC = − 1.14, *p* = 0.0198, Fig. 2A, Additional file 4: Table S4). Intriguingly, knockdown of miR-493-5p is associated with the hypermethylation of the MEG3 differentially regulated region (DMR) in liver cancer [12]. Therefore, miR-493-5p was selected for subsequent analysis. A positive correlation was noted between miR-493 and MEG3 expression in AML samples from TCGA (Fig. 2B). Then, after clinical sample collection, reduction in miR-493-5p was detected in AML bone marrow samples (Fig. 2C). In addition, a positive correlation between MEG3 and miR-493-5p expression was also seen in AML bone marrow samples (Fig. 2D).

Then, we observed reduced miR-493-5p expression in HL-60 and Molm13 cells in contrast to HS-5 cells, and it was lower in HL-60 R and Molm13 R cells than that in HL-60 and Molm13 cells. Silencing of MEG3 in AML parental cells significantly reduced the miR-493-5p expression, while overexpression of MEG3 in AML resistant cells upregulated miR-493-5p expression (Fig. 2E and Additional file 7: Fig. S2A).

Previous data have shown that miRNAs are closely related to chemoresistance in AML [21]. Here, we found reduced expression of miR-493-5p in AML drug-resistant cells. Therefore, we explored whether miR-493-5p expression was associated with AML cell growth and chemoresistance. AML parental cells and resistant cells were transfected with miR-493-5p inhibitor or mimic, respectively. miR-493-5p expression was decreased in HL-60 and Molm13 cells transfected with miR-493-5p inhibitor, while it increased in HL-60 R and Molm13 R cells transfected with miR-493-5p mimic (Fig. 2F and Additional file 7: Fig. S2B). In addition, the results of CCK-8 assay and flow cytometry (Fig. 2G, H and Additional file 7: Fig. S2C, D) exhibited suppressed apoptosis and enhanced viability in parental cells treated with AraC + miR-493-5p inhibitor, while promoted apoptosis and inhibited viability in resistant cells treated with AraC + miR-493-5p mimic. Additionally, Western blot analysis results (Fig. 2I and Additional file 7: Fig. S2E) presented that downregulation of miR-493-5p in parental cells reduced the levels of Bax and cleaved caspase-3 and increased Bcl-2 level, but upregulated miR-493-5p in resistant cells reversed these results, suggesting that intervention of miR-493-5p affected the sensitivity of AML cells to AraC.

Next focus of this study was on whether MEG3 enhances the sensitivity of AML cells to AraC by regulating miR-493-5p expression. RT-qPCR data (Fig. 2J and Additional file 7: Fig. S2F) displayed that miR-493-5p expression elevated in parental cells upon treatment with AraC + sh-MEG3 + miR-493-5p mimic, and it decreased in resistant cells upon treatment with AraC + oe-MEG3 + miR-493-5p inhibitor. Besides, the results of CCK-8 assay and flow cytometry (Fig. 2K, L and Additional file 7: Fig. S2G, H) exhibited that downregulated MEG3 and overexpressed miR-493-5p promoted apoptosis and inhibited viability of HL-60 and Molm13 cells treated with AraC, while these effects were counteracted after overexpression of MEG3 and inhibition of miR-493-5p in resistant cells. Western blot analysis results (Fig. 2M and Additional file 7: Fig. S2I) revealed that silencing of MEG3 and upregulation of miR-493-5p in parental cells increased the levels of Bax and cleaved caspase-3 and decreased Bcl-2 level, while overexpression of MEG3 and inhibition of miR-493-5p in resistant cells reversed these trends.

Cumulatively, MEG3 promoted the sensitivity of AML cells to AraC by elevating miR-493-5p expression.

### MiR-493-5p targets METTL3 and inhibits METTL3 expression

**Fig. 3:**
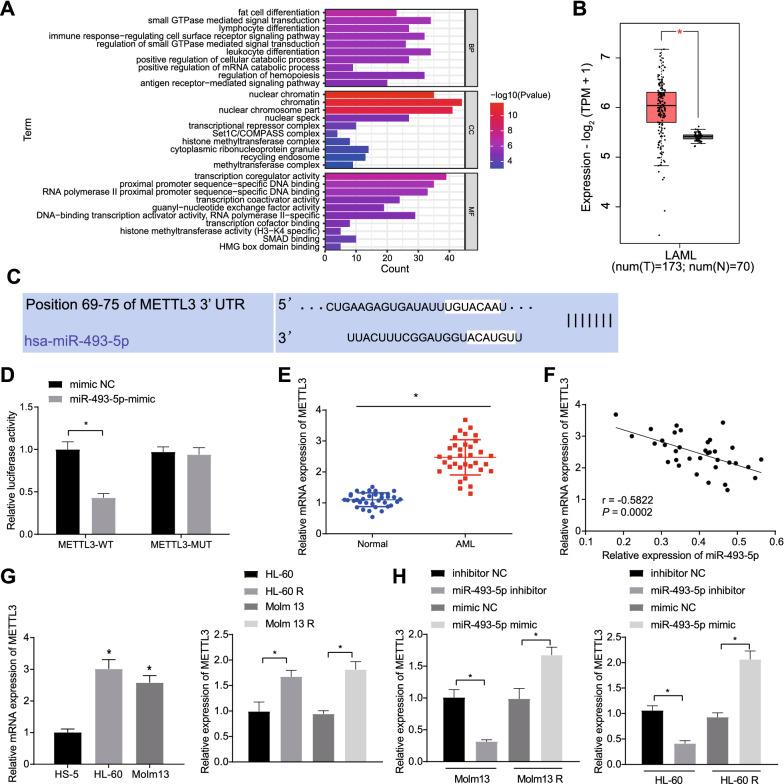
METTL3 is a target gene of miR-493-5p. A, GO functional enrichment analysis of the candidate genes. B, Differential expression of METTL3 in AML samples in TCGA and GTEx. The red box plot represents disease samples, and the gray box plot represents normal samples. C, starBase was used to predict the binding site of miR-493-5p to METTL3. D, Dual luciferase reporter assay was used to detect the binding of miR-493-5p to METTL3. E, METTL3 expression in bone marrow samples of AML patients (n = 35) and healthy individuals (n = 35) determined by RT-qPCR. F, Correlation analysis between miR-493-5p expression and METTL3 expression in the bone marrow samples of AML patients (n = 35). G, METTL3 expression in HS-5, HL-60 and Molm13 cells and HL-60 R and Molm13 R cells measured by RT-qPCR. H, METTL3 expression in HL-60 and Molm13 cells and HL-60 R and Molm13 R cells upon miR-493-5p inhibitor/mimic determined by RT-qPCR. * *p* < 0.05. Cell experiments were performed three times independently

GEPIA database was used to search DEGs in AML samples versus normal samples in TCGA and GTEx databases (Additional file 8: Fig. S3A). Also, the downstream genes of miR-493-5p predicted using the mirDIP and TargetScan databases were intersected with the DEGs obtained from TCGA database (Additional file 8: Fig. S3B), from which 582 candidate genes were identified (Additional file 5: Table S5). The GO functional enrichment analysis demonstrated that they were mainly enriched in GO terms such as “small GTPase mediated signal transduction” (Fig. 3A). Among these enriched genes, we found that METTL3 was enriched in T cell differentiation and methyltransferase complexes.

In addition, based on the results from TCGA and GTEx databases, METTL3 was found notably highly expressed in AML samples (Fig. 3B). Meanwhile, METTL3 has been documented to accelerate myeloid leukemia [16]. Therefore, we first investigated whether miR-493-5p could target METTL3 and regulate its expression. Based on the starBase database, a binding site was predicted between miR-493-5p and METTL3 (Fig. 3C), which was then confirmed by dual-luciferase assay (Fig. 3D). Also, increased METTL3 expression was detected in the bone marrow samples of AML patients, where miR-493-5p shared negative correlation with METTL3 expression (Fig. 3E, F).

Moreover, METTL3 was found highly expressed in HL-60 and Molm13 cells relative to HS-5 cells; meanwhile, METTL3 expression in HL-60 and Molm13 cells was lower than that in HL-60 R and Molm 13 R cells (Fig. 3G). Inhibition of miR-493-5p resulted in an increase of METTL3 in HL-60 and Molm13 cells, while overexpression of miR-493-5p caused a reduction in METTL3 expression in HL-60 R and Molm 13 R cells (Fig. 3H). Taken together, these data verified that miR-493-5p could target METTL3 and negatively regulated METTL3 expression.

### Sensitivity of AML cells to AraC is increased by miR-493-5p-mediated suppression of METTL3

**Fig. 4:**
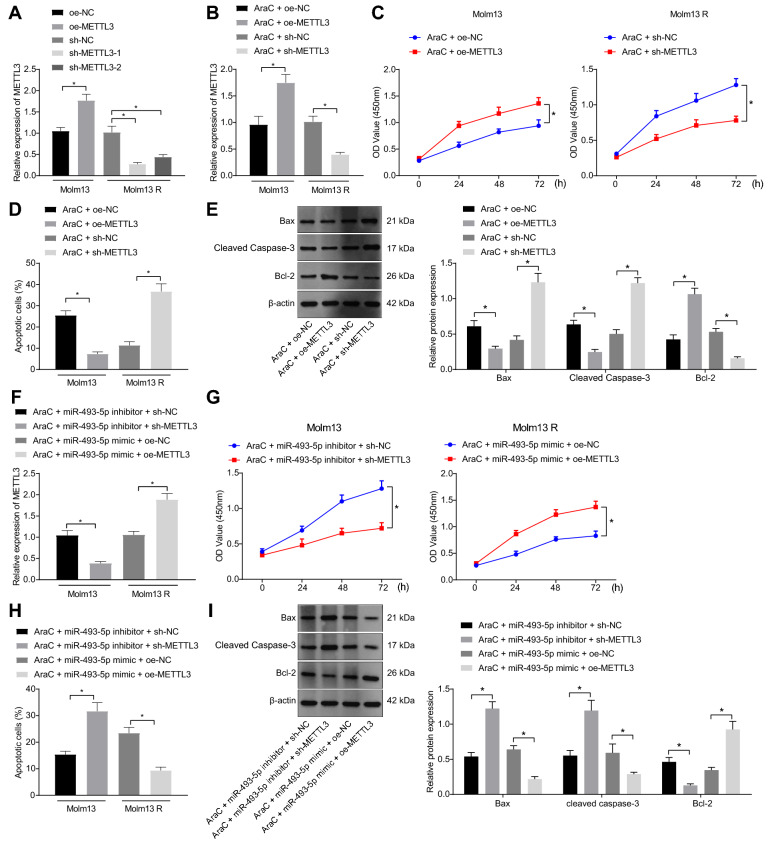
miR-493-5p promotes the sensitivity of Molm13 cells to AraC by targeting METTL3. A, The expression of METTL3 in Molm13 cells treated with oe-METTL3 and in Molm13 R cells treated with sh-METTL3-1 or sh-METTL3-2 measured by RT-qPCR. Molm13 cells were treated with AraC (2 μM) + oe-METTL, and Molm13 R cells were treated with AraC (2 μM) + sh-METTL. B, The expression of METTL3 in Molm13 and Molm13 R cells measured by RT-qPCR. C, The viability of Molm13 and Molm13 R cells evaluated using CCK-8 assay. D, The apoptosis of Molm13 and Molm13 R cells assessed using flow cytometry. E, The expression of Bcl-2, Bax, and cleaved caspase-3 proteins in Molm13 and Molm13 R cells determined by Western blot analysis. Molm13 cells were treated with AraC (2 μM) + miR-493-5p inhibitor + sh-METTL3, and Molm13 R cells were treated with AraC (2 μM) + miR-493-5p mimic + oe-METTL3. F, Determination of METTL3 expression in Molm13 and Molm13 R cells by RT-qPCR. G, Viability of Molm13 and Molm13 R cells assessed by CCK-8 assay. H, Apoptosis of Molm13 and Molm13 R cells determined by flow cytometry. I, Protein expression of Bcl-2, Bax, and cleaved caspase-3 in Molm13 and Molm13 R cells determined using Western blot analysis. * *p* < 0.05. Cell experiments were performed three times independently

A recent study has suggested that inhibition of METTL3 promotes chemoresistance of AML cells [22]. In this study, we then aimed to investigate the specific molecular mechanism of METTL3 affecting chemoresistance of AML cells. Both sh-METTL3-1 and sh-METTL3-2 could significantly inhibit METTL3 expression in AML cells (Fig. 4A and Additional file 9: Fig. S4A). Eventually, sh-METTL3-1 with the more markedly inhibited METTL3 expression was selected for subsequent experiments.

Next, the effects of METTL3 intervention on AML cells and drug sensitivity were further explored. RT-qPCR results exhibited increased METTL3 expression in HL-60 and Molm13 cells treated with AraC + oe-METTL3, but METTL3 expression was decreased in HL-60 R and Molm13 R cells treated with AraC + sh-METTL3 (Fig. 4B and Additional file 9: Fig. S4B). In addition, flow cytometry and CCK-8 assay results revealed that overexpression of METTL3 inhibited apoptosis and promoted viability of HL-60 and Molm13 cells treated with AraC, while silencing of METTL3 enhanced apoptosis and repressed viability of HL-60 R and Molm13 R cells treated with AraC (Fig. 4C, D and Additional file 9: Fig. S4C, D). Meanwhile, upregulated METTL3 reduced the expression of Bax and cleaved caspase-3 and elevated that of Bcl-2 in HL-60 and Molm13 cells treated with AraC, while these results were reversed in HL-60 R and Molm13 R cells co-treated with sh-METTL3 and AraC (Fig. 4E and Additional file 9: Fig. S4E).

RT-qPCR results showed that treatment with AraC + miR-493-5p inhibitor + sh-METTL3 reduced METTL3 expression in HL-60 and Molm13 cells while treatment with AraC + miR-493-5p mimic + oe-METTL3 elevated METTL3 expression in HL-60 R and Molm13 R cells (Fig. 4F and Additional file 9: Fig. S4F). Furthermore, after concomitant downregulation of miR-493-5p and METTL3, apoptosis was promoted, and viability was inhibited in HL-60 and Molm13 cells treated with AraC, while these trends were reversed following concomitant overexpression of miR-493-5p and METTL3 in HL-60 R and Molm13 R cells treated with AraC (Fig. 4G, H and Additional file 9: Fig. S4G, H). Moreover, Western blot analysis results displayed that miR-493-5p and METTL3 knockdown elevated levels of Bax and cleaved caspase-3 and reduced Bcl-2 in HL-60 and Molm13 cells, while these results were reversed by concomitant overexpression of miR-493-5p and METTL3 in HL-60 R and Molm13 R cells treated with AraC (Fig. 4I and Additional file 9: Fig. S4I). Collectively, miR-493-5p enhanced the sensitivity of AML cells to AraC by suppressing METTL3 expression.

### METTL3 promotes MYC expression through MYC m6A methylation to increase resistance of AML cells to AraC

**Fig. 5:**
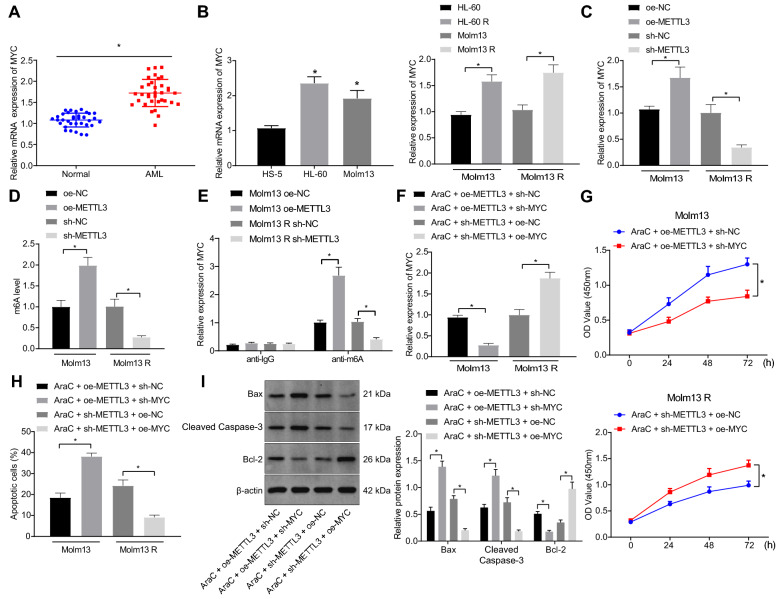
METTL3 upregulates MYC expression through MYC m6A methylation and reduces the sensitivity of Molm13 cells to AraC. A, MYC expression in the bone marrow samples of AML patients (n = 35) and healthy individuals (n = 35) determined by RT-qPCR. B, MYC expression in HS-5, HL-60, Molm13, HL-60 R, and Molm13 R cells measured by RT-qPCR. Molm13 cells were treated with oe-METTL, and Molm13 R cells were treated with sh-METTL. C, MYC expression in Molm13 and Molm13 R cells measured by RT-qPCR. D, The m6A modification level in Molm13 and Molm13 R cells with methylene blue staining as control detected by Dot blot assay. E, Me-RIP and RT-qPCR quantification of the MYC-modified m6A level. Molm13 cells were treated with AraC (2 μM) + oe-METTL3 + sh-MYC, and Molm13 R cells were treated with AraC (2 μM) + sh-METTL3 + oe-MYC. F, MYC expression in Molm13 and Molm13 R cells measured by RT-qPCR. G, Viability of Molm13 and Molm13 R cells evaluated by CCK-8 assay. H, Apoptosis of Molm13 and Molm13 R cells detected by flow cytometry. I, Expression of Bcl-2, Bax, and cleaved caspase-3 protein in Molm13 and Molm13 R cells determined using Western blot analysis. * *p* < 0.05. Cell experiments were performed three times independently

METTL3 can induce m6A modification of MYC mRNA to promote its expression [23] and MYC overexpression increases the chemoresistance of AML cells [24]. We then focused on whether the regulatory role of METTL3 in AML was achieved through mediation of MYC. We identified an increase in MYC mRNA expression in the bone marrow samples of AML patients (Fig. 5A) as well as in HL-60 and Molm13 cells (Fig. 5B). Moreover, MYC mRNA expression was higher in HL-60 R and Molm13 R cells than that in HL-60 and Molm13 cells (Fig. 5B).

Upregulated METTL3 elevated MYC mRNA expression in HL-60 and Molm13 cells, and inhibition of METTL3 in HL-60 R and Molm13 R cells remarkably suppressed MYC mRNA expression (Fig. 5C and Additional file 10: Fig. S5A). Dot blot assays and Me-RIP-RT-qPCR both exhibited that overexpressed METTL3 in HL-60 and Molm13 cells elevated m6A level and m6A-modified MYC mRNA level, while these effects were negated by silencing of METTL3 in HL-60 R and Molm13 R cells (Fig. 5D, E and Additional file 10: Fig. S5B, C).

Treatment with AraC + oe-METTL3 + sh-MYC in HL-60 and Molm13 cells reduced MYC expression, while AraC + sh-METTL3 + oe-MYC treatment in HL-60 R and Molm13 R cells elevated MYC expression (Fig. 5F and Additional file 10: Fig. S5D). In addition, AraC + oe-METTL3 + sh-MYC treatment enhanced HL-60 and Molm13 cell apoptosis but inhibited cell viability as evidenced by elevated expression of pro-apoptosis genes and decreased anti-apoptosis gene expression while these effects were abolished by AraC + sh-METTL3 + oe-MYC in HL-60 R and Molm13 R cells (Fig. 5G–I and Additional file 10: Fig. S5E-G). Together, these findings showed that METTL3 promoted MYC expression by regulating MYC m6A methylation and reduced the sensitivity of AML cells to AraC.

### MEG3 promotes the sensitivity of AML cells to AraC through inhibition of the METTL3/MYC axis by upregulating miR-493-5p

**Fig. 6:**
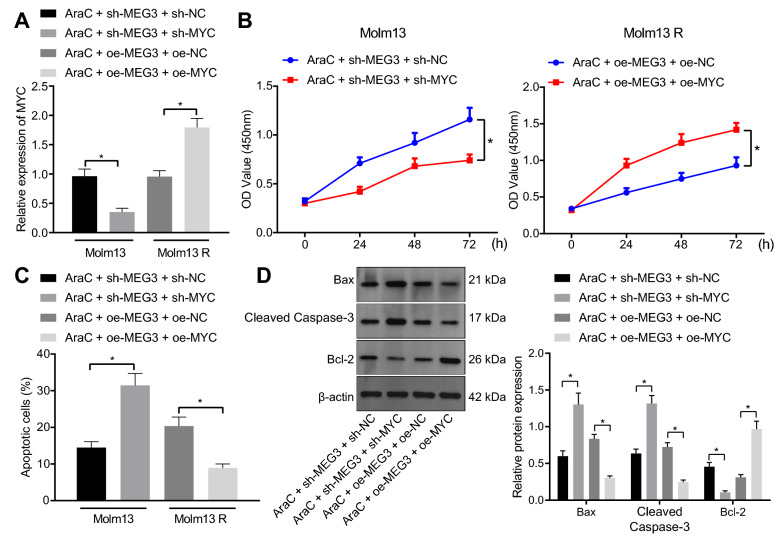
MEG3 upregulates miR-493-5p and downregulates the METTL3/MYC axis to promote the sensitivity of Molm13 cells to AraC. Molm13 cells were treated with AraC (2 μM) + sh-MEG3 + sh-MYC, and Molm13 R cells were treated with AraC (2 μM) + oe-MEG3 + oe-MYC. A, MYC expression in Molm13 and Molm13 R cells determined using RT-qPCR. B, The viability of Molm13 and Molm13 R cells evaluated by CCK-8 assay. C, Apoptosis of Molm13 nd Molm13 R cells detected by flow cytometry. D, Expression of Bcl-2, Bax, and cleaved caspase-3 proteins in Molm13 and Molm13 R cells determined using Western blot analysis. * *p* < 0.05. Cell experiments were performed three times independently

The aforementioned results allowed us to characterize whether MEG3 enhances the sensitivity of AML cells to AraC through regulation of the miR-493-5p/METTL3/MYC axis. RT-qPCR data revealed that treatment with AraC + sh-MEG3 + sh-MYC reduced MYC expression in HL-60 and Molm13 cells while an opposite result was noted following AraC + oe-MEG3 + oe-MYC in HL-60 R and Molm13 R cells (Fig. 6A and Additional file 11: Fig. S6A).

Subsequently, both silencing of MEG3 and MYC promoted cell apoptosis, weakened cell viability, increased levels of pro-apoptosis genes, and decreased anti-apoptosis gene level in HL-60 and Molm13 cells treated with AraC. However, these results were reversed by both overexpression of MEG3 and MYC in HL-60 R and Molm13 R cells treated with AraC (Fig. 6B–D and Additional file 11: Fig. S6B–D). In conclusion, these findings indicated that MEG3 downregulated the METTL3/MYC axis by promoting miR-493-5p expression to promote the sensitivity of AML cells to AraC.

### *MEG3 suppresses the resistance of AML cells to AraC *in vivo

Finally, we shifted our attention to validate the effect of MEG3 on the resistance of AML cells to AraC in vivo. The intensity was weakened in mice injected with Molm13-luc-GFP AML cells with depleted MEG3 or Molm13 R-luc-GFP AML cells overexpressing MEG3 and/or treated with AraC.

**Fig. 7:**
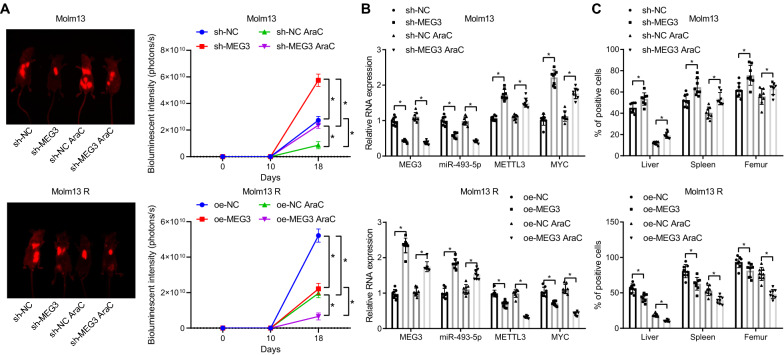
MEG3 promotes the sensitivity of AML cells to AraC in vivo. A, Quantitative analysis of bioluminescence imaging to determine the luminescence intensity. B, The expression of MEG3, miR-493-5p, METTL3 and MYC in the bone marrow samples of mice determined by RT-qPCR. C, Content of AML cells in liver, spleen, and femur tissues of mice detected by IHC. * *p* < 0.05. n = 8

It was found that silencing of MEG3 promoted bioluminescence intensity in Molm13 cells, treatment with AraC relieved bioluminescence intensity, and silencing of MEG3 combined with AraC enhanced bioluminescence intensity. Moreover, bioluminescence intensity was repressed in mice injected with Molm13 R cells overexpressing MEG3, and it was also inhibited after further treatment with AraC (Fig. 7A).

In order to further observe whether MEG3 affects the expression of miR-493-5p, METTL3 and MYC, mouse bone marrow samples were taken for detection. RT-qPCR showed that mice with Molm13 cells expressing reduced MEG3 limited expression of MEG3 and miR-493-5p, and elevated expression METTL3 and MYC, while injection of Molm13 R cells overexpressing MEG3 counterweighed these effects (Fig. 7B).

Human leukemia cells were specifically identified by IHC with anti-firefly fluorescent antibody (Fig. 7C, Additional file 12: Fig. S7), which displayed that AraC treatment reduced the burden of leukemia. After injection of Molm13 cells with MEG3 knockdown, the sensitivity to AraC decreased, while after injection of Molm13 R cells overexpressing MEG3, their sensitivity to AraC increased in mice.

Thus, MEG3 promotes the sensitivity of AML cells to AraC and increases survival rate by regulating the miR-493-5p/METTL3/MYC axis in vivo.

## Discussion

AML is a complicated disease attributed to the considerable molecular diversity of AML, the older age of the majority of AML patients, and, more commonly, resistance to treatment [25]. Targeting the resistance to treatment, efforts are increasingly directed towards increasing sensitivity to chemotherapy, among which analysis of the lncRNA-miRNA regulatory network can help identify molecules of prognostic or therapeutic relevance in AML [26]. Our study thus focused on the regulatory network of MEG3/miR-493-5p/METTL3 in AML, aiming to determine a target pathway that could potentially be leveraged to enhance the sensitivity of AML cells to chemotherapy.

As a prior study showed, an inhibitory effect of MEG3 on the onset and progression of AML has been identified [20]. Low expression of MEG3 has been detected in AML cells [27]. Consistently, we also found that MEG3 was poorly expressed in AML cells and tissues and that MEG3 overexpression could induce the apoptosis of AML cells while repressing their viability. It was shown that overexpressed MEG3 could elevate the expression of Bax, and cleaved caspase-3 but reduce that of Bcl-2, further demonstrating its pro-apoptotic effect. Bax, and cleaved caspase-3 are known as pro-apoptosis markers while Bcl-2 is considered as an anti-apoptosis marker [28]. Furthermore, we found that overexpressed MEG3 could promote the sensitivity of AML cells to AraC. AraC is the key element of all cytostatic AML treatments [29]. The theoretical basis for AraC therapy was developed in the 1970s and has since been utilized in AML treatment [30]. The findings of this study demonstrated that overexpressed MEG3 and AraC facilitated the apoptosis of AML cells treated with AraC.

Furthermore, the pertinence between MEG3 and miR-493-5p in AML was studied with positive relation found. Such correlation has been reported earlier, where the silencing of miR-493-5p was related to MEG3-DMR hypermethylation [12]. In addition, MEG3 can inhibit the proliferation of neural stem cells after ischemic stroke via positive regulation of miR-493-5p [31]. However, interactions between MEG3 and miR-493-5p impacting AML cells have not been identified before. Our study proposed that overexpressed MEG3 promoted miR-493-5p expression to further heighten the sensitivity of AML cells to AraC. Interestingly, the depletion of miR-493 is capable of inducing the resistance to cisplatin in lung cancer cells by TCRP1 [32]. Consistently, here we found that the inhibition of miR-493 could reverse the promotive effects of MEG3 on the sensitivity of AML cells to AraC by restraining AML cell apoptosis.

Data collected from the starBase database and luciferase reporter assay revealed that miR-493-5p might target METTL3 and downregulate its expression. Emerging evidence demonstrates that miRNAs can interact with the 3’UTR of specific target mRNAs and subsequently result in inhibition of their expression [33, 34]. This represents the first evidence for the post-transcriptional regulation of METTL3 by miR-493-5p in AML cells and might have importance in regulating AML progression. METTL3 is able to mediate myeloid differentiation of leukemia cells [16]. Increased sensitivity to anticancer reagents including 5-fluorouracil and cisplatin, and irradiation has been observed in METTL3-depleted pancreatic cancer cells [35], which is consistent with our findings showing that METTL3 knockdown could enhance the sensitivity of AML cells to chemotherapy. This study also demonstrated that METTL3 promoted MYC expression through MYC m6A methylation. METTL3 can induce m6A modification of MYC mRNA and consequently increases its expression in bladder cancer [23]. Overexpression of MYC has been demonstrated to exacerbate the resistance of AML cells to chemotherapy [24], which is in line with our findings. Conclusively, we reasoned that the loss of miR-493-5p could elevate METTL3 expression, which promoted MYC expression and, furthermore, the resistance of AML cells to AraC. This finding was validated in the AML cells by performing rescue experiments. Finally, animal experiments substantiated the anti-tumor effect of MEG3 and its promotive effect on the sensitivity to chemotherapy. It was also verified that MEG3 exerted an effect through miR-493-5p-dependent inhibition of METTL3 and subsequent suppression of MYC.

## Conclusion

**Fig. 8:**
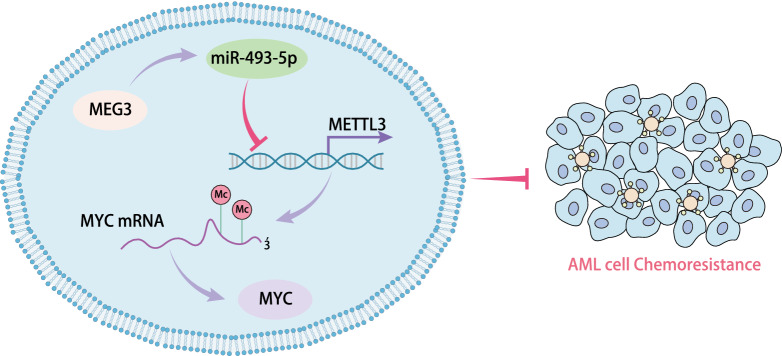
Diagram of molecular mechanism. MEG3 promotes the expression of miR-493-5p which targets and suppresses METTL3, resulting in attenuation of METTL3 on MYC mRNA m6A methylation modification and reduction of MYC expression, eventually increasing the sensitivity of AML cells to AraC

Taken together, MEG3 can upregulate the expression of miR-493-5p which targets and suppresses METTL3, reducing the role of METTL3 on MYC mRNA m6A methylation modification and downregulating MYC expression. By this mechanism, MEG3 increases the sensitivity of AML cells to AraC (Fig. 8). The potential of the lncRNA-miRNA regulatory networks as molecular targets is still under exploration. Still, further studies with larger sample size for control are needed to confirm the clinical application of the biomarker of MEG3 in the chemotherapy of AML. In addition, further investigation is also required on whether METTL3-mediated MYC mRNA m6A methylation modification affects the half-life and stability of MYC mRNA, and whether some para-signaling pathways participate in the inhibitory role of MEG3 in the chemoresistance of AML through the miR-493-5p/METTL3/MYC axis.

## Supplementary Information

**Additional file 1: Table S1** Clinical characteristics of AML patients and the related controls

**Additional file 2: Table S2** shRNA sequences

**Additional file 3: Table S3** Primer sequences for RT-qPCR

**Additional file 4: Table S4** Differentially expressed genes in chip GSE62137

**Additional file 5: Table S5** 582 candidate target genes

**Additional file 6: Figure S1** Restored MEG3 stimulates the apoptosis of HL-60 cells and increases the sensitivity of HL-60 cells to AraC. **A** The expression of MEG3 in HL-60 and HL-60 R cells following treatment with AraC at different concentrations determined by RT-qPCR. **B** The viability of HL-60 and HL-60 R cells following treatment with AraC at different concentrations measured by CCK-8 assay. **C** The transfection efficiency of MEG3 in HL-60 and HL-60 R cells detected by RT-qPCR. **D** The viability of HL-60 cells co-treated with 2 μM AraC and sh-MEG3 and HL-60 R cells co-treated with 2 μM AraC and oe-MEG3 measured by CCK-8 assay. **E** Apoptosis of HL-60 cells co-treated with 2 μM AraC and sh-MEG3 and HL-60 R cells co-treated with 2 μM AraC and oe-MEG3 determined by flow cytometry. **F** The expression of apoptosis-related proteins Bcl-2, Bax, and cleaved caspase-3 in HL-60 cells co-treated with 2 μM AraC and sh-MEG3 and HL-60 R cells co-treated with 2 μM AraC and oe-MEG3 determined by Western blot analysis. * *p* < 0.05. Cell experiments were performed three times independently

**Additional file 7: Figure S2** MEG3 upregulates miR-493-5p to strengthen the sensitivity of HL-60 cells to AraC. **A** miR-493-5p expression in HL-60 and HL-60 R cells quantified by RT-qPCR. HL-60 cells were treated with AraC (2 μM) + miR-493-5p inhibitor and HL-60 R cells were treated with AraC (2 μM) + miR-493-5p mimic. **B** miR-493-5p expression in HL-60 and HL-60 R cells determined by RT-qPCR. **C** Viability of HL-60 and HL-60 R cells determined by CCK-8 assay. **D** Apoptosis of HL-60 and HL-60 R cells determined by flow cytometry. HL-60 cells were treated with AraC (2 μM) + sh-MEG3 + miR-493-5p mimic and HL-60 R cells were treated with AraC (2 μM) + oe-MEG3 + miR-493-5p inhibitor. **E** The expression of Bcl-2, Bax, and cleaved caspase-3 proteins in the HL-60 and HL-60 R cells determined by Western blot analysis. **F** miR-493-5p expression in the HL-60 and HL-60 R cells determined by RT-qPCR. **G** Viability of HL-60 and HL-60 R cells determined by CCK-8 assay. **H** Apoptosis of HL-60 and HL-60 R cells determined by flow cytometry. **I** The expression of Bcl-2, Bax, and cleaved caspase-3 proteins in HL-60 and HL-60 R cells determined by Western blot analysis. * p < 0.05. Cell experiments were performed three times independently

**Additional file 8: Figure S3** A heat map obtained using bioinformatics analysis for prediction of miR-493-5p target genes. **A** A heat map depicting the differential gene expression between normal samples and AML samples in TCGA and GTEx. The red short line indicates highly expressed genes in AML samples, the green short line indicates poorly expressed genes in AML samples, and the location of the short line indicates the location of this gene on the chromosome. **B** Prediction of miR-493-5p target genes by mirDIP and TargetScan databases. The three circles indicate prediction results using the two databases, respectively, and the significant highly expressed genes in AML samples in TCGA and GTEx predicted by GEPIA. The central part indicates the intersection of the three groups of data

**Additional file 9: Figure S4** miR-493-5p promotes the sensitivity of HL-60 cells to AraC by targeting METTL3. **A** METTL3 expression in HL-60 cells treated with oe-METTL3 and in HL-60 R cells treated with sh-METTL3-1 or sh-METTL3-2 determined by RT-qPCR. HL-60 cells were treated with AraC (2 μM) + oe-METTL, and HL-60 R cells were treated with AraC (2 μM) + sh-METTL. **B** METTL3 expression in HL-60 and HL-60 R cells determined by RT-qPCR. **C** Viability of HL-60 and HL-60 R cells determined by CCK-8 assay. **D** Apoptosis of HL-60 and HL-60 R cells determined by flow cytometry. **E** The expression of Bcl-2, Bax, and cleaved caspase-3 proteins in HL-60 and HL-60 R cells determined by Western blot analysis. HL-60 cells were treated with AraC (2 μM) + miR-493-5p inhibitor + sh-METTL3, and HL-60 R cells were treated with AraC (2 μM) + miR-493-5p mimic + oe-METTL3. **F** METTL3 expression in HL-60 and HL-60 R cells determined by Western blot analysis. **G** Viability of HL-60 and HL-60 R cells determined by CCK-8 assay. **H** Apoptosis of HL-60 and HL-60 R cells determined by flow cytometry. **I** The expression of Bcl-2, Bax, and cleaved caspase-3 proteins in HL-60 and HL-60 R cells determined by Western blot analysis. * *p* < 0.05. Cell experiments were performed three times independently.

**Additional file 10: Figure S5** METTL3 upregulates MYC expression through MYC m6A methylation and reduces the sensitivity of HL-60 cells to AraC. HL-60 cells were transfected with oe-METTL3 and HL-60 R cells were transfected with sh-METTL3. A MYC expression in HL-60 and HL-60 R cells measured by RT-qPCR. B The m6A modification level in Molm13 and Molm13 R cells with methylene blue staining as control detected by Dot blot assay. C Me-RIP and RT-qPCR quantification of the MYC-modified m6A level. HL-60 cells were treated with AraC (2 μM) + oe-METTL3 + sh-MYC, and HL-60 R cells were treated with AraC (2 μM) + sh-METTL3 + oe-MYC. D MYC expression in HL-60 and HL-60 R cells measured by RT-qPCR. E Viability of HL-60 and HL-60 R cells evaluated by CCK-8 assay. F Apoptosis of HL-60 and HL-60 R cells detected by flow cytometry. G Expression of Bcl-2, Bax, and cleaved caspase-3 proteins in HL-60 and HL-60 R cells determined using Western blot analysis.* *p* < 0.05. Cell experiments were performed three times independently

**Additional file 11: Figure S6 **MEG3 upregulates miR-493-5p and downregulates the METTL3/MYC axis to promote the sensitivity of HL-60 cells to AraC. HL-60 cells were treated with AraC (2 μM) + sh-MEG3 + sh-MYC, and HL-60 R cells were treated with AraC (2 μM) + oe-MEG3 + oe-MYC. A MYC expression in HL-60 and HL-60 R cells was determined by RT-qPCR. B Viability of HL-60 and HL-60 R cells was determined by CCK-8 assay. C Apoptosis of HL-60 and HL-60 R cells was detected by flow cytometry. D The expression of Bcl-2, Bax, and cleaved caspase-3 proteins in HL-60 and HL-60 R cells determined by Western blot analysis. * *p* < 0.05. Cell experiments were performed three times independently

**Additional file 12: Figure S7** Representative IHC images of the content of AML cells in liver, spleen, and femur tissues of mice

## Data Availability

Data will be made available on reasonable request.
