# Supplementary material for: Tumor-suppressive MEG3 induces microRNA-493-5p expression to reduce arabinocytosine chemoresistance of acute myeloid leukemia cells by downregulating the METTL3/MYC axis
Source: J Transl Med. 2022 Jun 27;20:288. doi: 10.1186/s12967-022-03456-x (PMC9235226; doi:10.1186/s12967-022-03456-x)
Supplement: Supplementary file 1 — Additional file 1: Table S1 Clinical characteristics of AML patients and the related controls [file 12967_2022_3456_MOESM1_ESM.doc]

**Table S1** Clinical characteristics of AML patients and the related controls

| Characteristics | Control (n = 35) | AML (n = 35) |
| --- | --- | --- |
| Sex (male/female) | 24/11 | 20/15 |
| Age (years) | 14-72 | 21-79 |
| WBC (×10^9^/L) | 5.25 ± 1.06 | 25.36 ± 9.42 |
| HGB (g/dL) | 17.13 ± 3.82 | 8.34 ± 2.01 |
| PLT (×10^9^/L) | 251.44 ± 18.95 | 52.48 ± 9.63 |
| LDH (U/L) | 213.67 ± 26.43 | 407.02 ± 32.56 |
| Present FAB subtype |  |  |
| M0 | NA | 2 |
| M1 | NA | 5 |
| M2 | NA | 4 |
| M3 | NA | 9 |
| M4 | NA | 8 |
| M5 | NA | 5 |
| M6 | NA | 2 |
| Molecular subtype |  |  |
| FLT3-ITDneg/NPM1mut | NA | 16 |
| FLT3-ITDpos/NPM1mut | NA | 6 |
| FLT3-ITDneg/NPM1^WT^ | NA | 4 |
| FLT3-ITDpos/NPM1^WT^ | NA | 3 |

Note: AML, acute myeloid leukemia; WBC, white blood cells; HGB, hemoglobin; PLT, platelets; LDH, lactate dehydrogenase; FAB, French-American-Britain; NA, no answer; FLT3-ITDneg, FMS-like tyrosine kinase 3 internal tandem duplication-negative; NPM1mut, nucleophosmin mutant.
