# Supplementary material for: Tumor-suppressive MEG3 induces microRNA-493-5p expression to reduce arabinocytosine chemoresistance of acute myeloid leukemia cells by downregulating the METTL3/MYC axis
Source: J Transl Med. 2022 Jun 27;20:288. doi: 10.1186/s12967-022-03456-x (PMC9235226; doi:10.1186/s12967-022-03456-x)
Supplement: Supplementary file 2 — Additional file 2: Table S2 shRNA sequences [file 12967_2022_3456_MOESM2_ESM.doc]

**Table S2 shRNA sequences**

| shRNA | Sequence (5’-3’) |
| --- | --- |
| sh-NC | UUCUCCGAACGUGUCACGUTT |
| sh-MEG3-1 | GGAAGAGGCTGCAGACGTTAA |
| sh-MEG3-2 | GAAGAGGCTGCAGACGTTAAT |
| sh-MYC-1 | CATTGGCTCTTCTCAAGCTCT |
| sh-MYC-1 | CGAGGACATCTGGAAGAAATT |
| sh-METTL3-1 | GCCTTAACATTGCCCACTGAT |
| sh-METTL3-2 | GCAAGTATGTTCACTATGAAA |

Note: sh, short hairpin; NC, negative control; MEG3, maternally expressed gene 3; METTL3, methyltransferase-like 3.
