# Supplementary material for: Tumor-suppressive MEG3 induces microRNA-493-5p expression to reduce arabinocytosine chemoresistance of acute myeloid leukemia cells by downregulating the METTL3/MYC axis
Source: J Transl Med. 2022 Jun 27;20:288. doi: 10.1186/s12967-022-03456-x (PMC9235226; doi:10.1186/s12967-022-03456-x)
Supplement: Supplementary file 3 — Additional file 3: Table S3 Primer sequences for RT-qPCR [file 12967_2022_3456_MOESM3_ESM.doc]

**Table S3** Primer sequences for RT-qPCR

| Target | Sequences |
| --- | --- |
| MEG3 | F: 5'-TGGGACCCCAGCCCCTCTCCAGC-3' |
|  | R: 5'-CCGGGTCCAGAGTCTCTGGG-3' |
| METTL3 | F: 5'-CAAGCTGCACTTCAGACGAA-3' |
|  | R: 5'-GCTTGGCGTGTGGTCTTT-3' |
| miR-493-5p | F: 5'-TTGTACATGGTAGGCTTTCATT-3' |
|  | R: Universal reverse primer |
| MYC | F: 5'-ATTGGTCAAGTCGGCCAGA-3' |
|  | F: 5'-TTCGGGTAGTGGAAAACCAG-3' |
|  | R: 5'-AGTAGAAATACGGCTGCACC-3' |
| β-actin | F: 5'-CCTTGCACATGCCGGAG-3' |
|  | R: 5'-GCACAGAGCCTCGCCTT-3' |
| U6 | F: 5'-GCTTCGGCAGCACATATACTAAAAT-3' |
|  | R: Universal reverse primer |

Note: RT-qPCR: Reverse transcription quantitative polymerase chain reaction; F: forward; R: reverse; MEG3, maternally expressed gene 3; METTL3, methyltransferase-like 3; miR-493-5p, microRNA-493-5p.
