# Supplementary figures and images for: Tumor-suppressive MEG3 induces microRNA-493-5p expression to reduce arabinocytosine chemoresistance of acute myeloid leukemia cells by downregulating the METTL3/MYC axis
Source: J Transl Med. 2022 Jun 27;20:288. doi: 10.1186/s12967-022-03456-x (PMC9235226; doi:10.1186/s12967-022-03456-x)

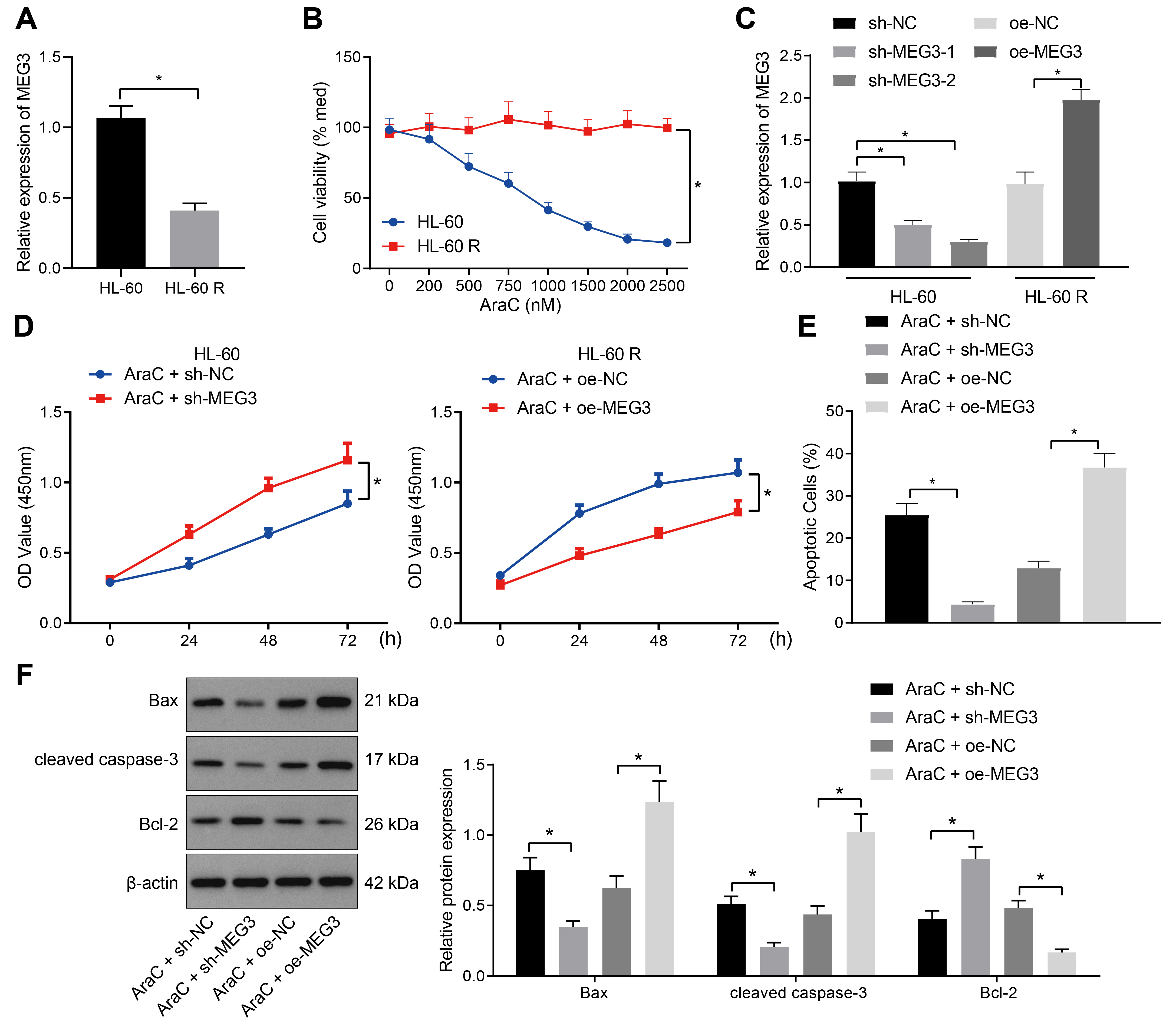

Supplement: Supplementary file 6 — Additional file 6: Figure S1 Restored MEG3 stimulates the apoptosis of HL-60 cells and increases the sensitivity of HL-60 cells to AraC. A The expression of MEG3 in HL-60 and HL-60 R cells following treatment with AraC at different concentrations determined by RT-qPCR. B The viability of HL-60 and HL-60 R cells following treatment with AraC at different concentrations measured by CCK-8 assay. C The transfection efficiency of MEG3 in HL-60 and HL-60 R cells detected by RT-qPCR. D The viability of HL-60 cells co-treated with 2 μM AraC and sh-MEG3 and HL-60 R cells co-treated with 2 μM AraC and oe-MEG3 measured by CCK-8 assay. E Apoptosis of HL-60 cells co-treated with 2 μM AraC and sh-MEG3 and HL-60 R cells co-treated with 2 μM AraC and oe-MEG3 determined by flow cytometry. F The expression of apoptosis-related proteins Bcl-2, Bax, and cleaved caspase-3 in HL-60 cells co-treated with 2 μM AraC and sh-MEG3 and HL-60 R cells co-treated with 2 μM AraC and oe-MEG3 determined by Western blot analysis. * p < 0.05. Cell experiments were performed three times independently [file 12967_2022_3456_MOESM6_ESM.jpg]

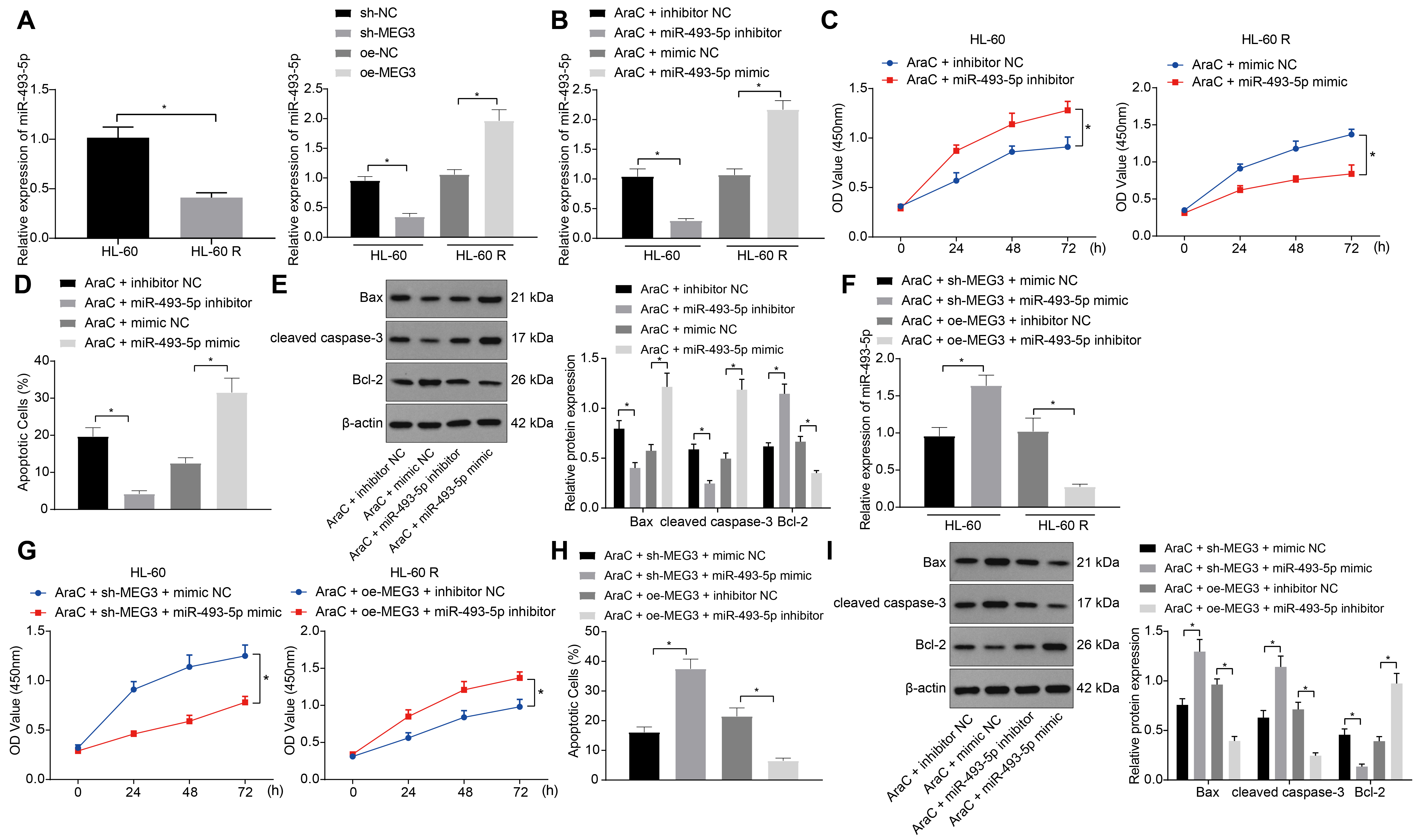

Supplement: Supplementary file 7 — Additional file 7: Figure S2 MEG3 upregulates miR-493-5p to strengthen the sensitivity of HL-60 cells to AraC. A miR-493-5p expression in HL-60 and HL-60 R cells quantified by RT-qPCR. HL-60 cells were treated with AraC (2 μM) + miR-493-5p inhibitor and HL-60 R cells were treated with AraC (2 μM) + miR-493-5p mimic. B miR-493-5p expression in HL-60 and HL-60 R cells determined by RT-qPCR. C Viability of HL-60 and HL-60 R cells determined by CCK-8 assay. D Apoptosis of HL-60 and HL-60 R cells determined by flow cytometry. HL-60 cells were treated with AraC (2 μM) + sh-MEG3 + miR-493-5p mimic and HL-60 R cells were treated with AraC (2 μM) + oe-MEG3 + miR-493-5p inhibitor. E The expression of Bcl-2, Bax, and cleaved caspase-3 proteins in the HL-60 and HL-60 R cells determined by Western blot analysis. F miR-493-5p expression in the HL-60 and HL-60 R cells determined by RT-qPCR. G Viability of HL-60 and HL-60 R cells determined by CCK-8 assay. H Apoptosis of HL-60 and HL-60 R cells determined by flow cytometry. I The expression of Bcl-2, Bax, and cleaved caspase-3 proteins in HL-60 and HL-60 R cells determined by Western blot analysis. * p < 0.05. Cell experiments were performed three times independently [file 12967_2022_3456_MOESM7_ESM.jpg]

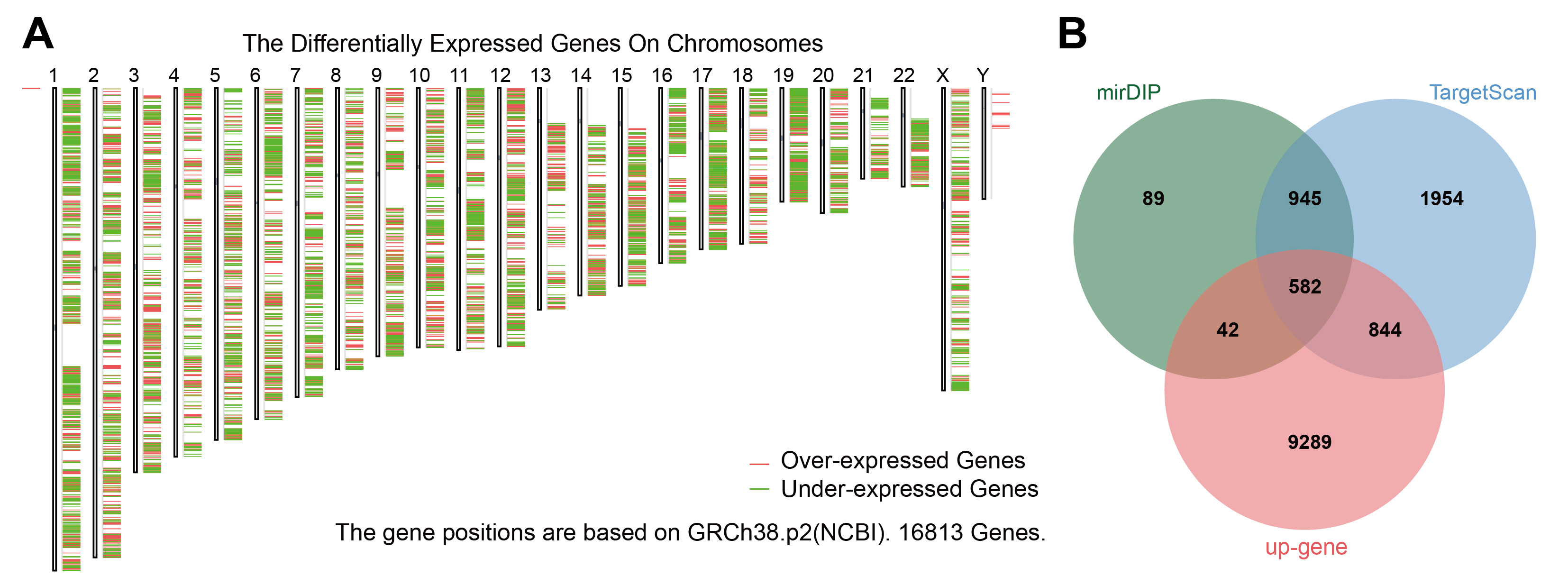

Supplement: Supplementary file 8 — Additional file 8: Figure S3 A heat map obtained using bioinformatics analysis for prediction of miR-493-5p target genes. A A heat map depicting the differential gene expression between normal samples and AML samples in TCGA and GTEx. The red short line indicates highly expressed genes in AML samples, the green short line indicates poorly expressed genes in AML samples, and the location of the short line indicates the location of this gene on the chromosome. B Prediction of miR-493-5p target genes by mirDIP and TargetScan databases. The three circles indicate prediction results using the two databases, respectively, and the significant highly expressed genes in AML samples in TCGA and GTEx predicted by GEPIA. The central part indicates the intersection of the three groups of data [file 12967_2022_3456_MOESM8_ESM.jpg]

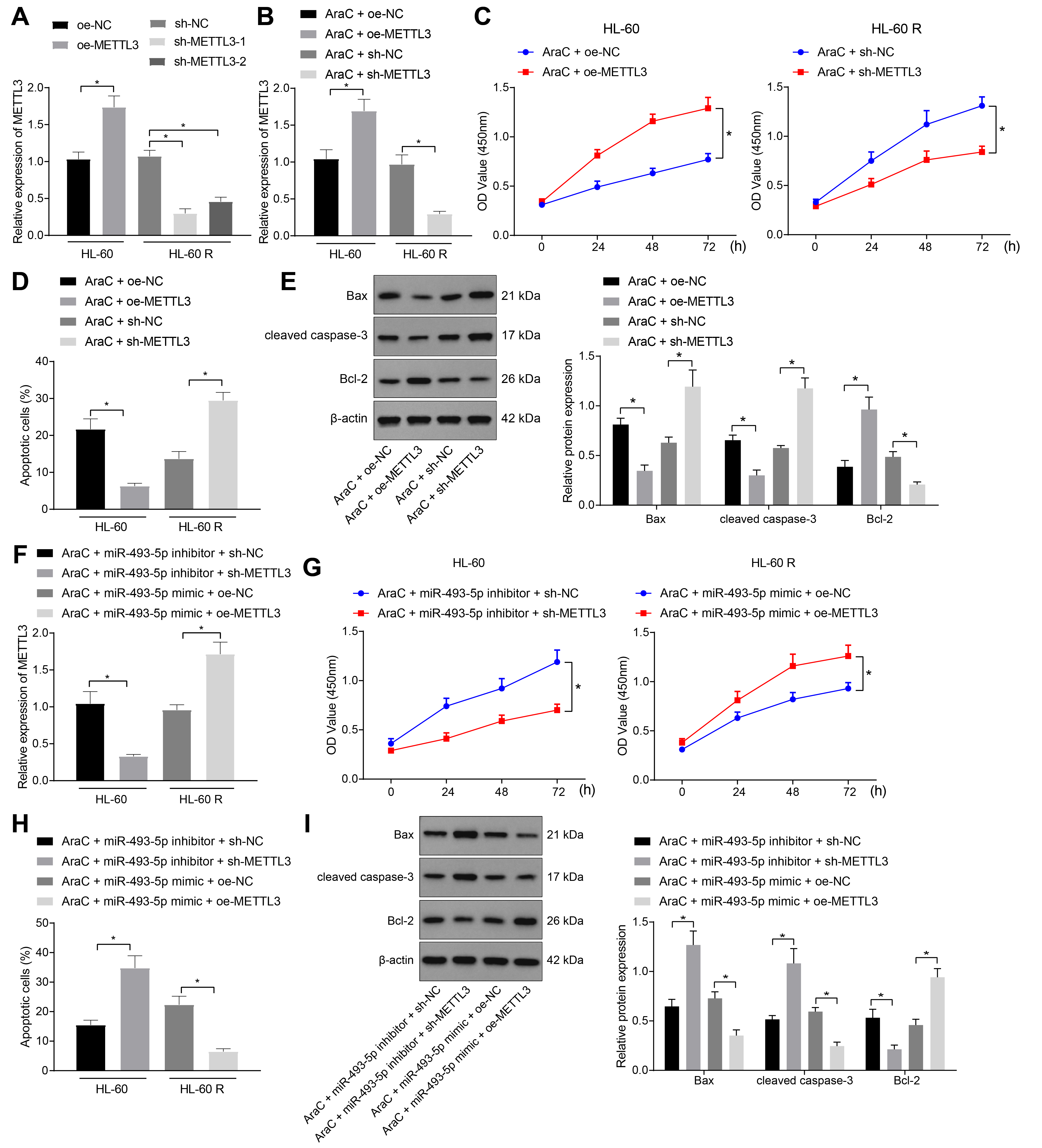

Supplement: Supplementary file 9 — Additional file 9: Figure S4 miR-493-5p promotes the sensitivity of HL-60 cells to AraC by targeting METTL3. A METTL3 expression in HL-60 cells treated with oe-METTL3 and in HL-60 R cells treated with sh-METTL3-1 or sh-METTL3-2 determined by RT-qPCR. HL-60 cells were treated with AraC (2 μM) + oe-METTL, and HL-60 R cells were treated with AraC (2 μM) + sh-METTL. B METTL3 expression in HL-60 and HL-60 R cells determined by RT-qPCR. C Viability of HL-60 and HL-60 R cells determined by CCK-8 assay. D Apoptosis of HL-60 and HL-60 R cells determined by flow cytometry. E The expression of Bcl-2, Bax, and cleaved caspase-3 proteins in HL-60 and HL-60 R cells determined by Western blot analysis. HL-60 cells were treated with AraC (2 μM) + miR-493-5p inhibitor + sh-METTL3, and HL-60 R cells were treated with AraC (2 μM) + miR-493-5p mimic + oe-METTL3. F METTL3 expression in HL-60 and HL-60 R cells determined by Western blot analysis. G Viability of HL-60 and HL-60 R cells determined by CCK-8 assay. H Apoptosis of HL-60 and HL-60 R cells determined by flow cytometry. I The expression of Bcl-2, Bax, and cleaved caspase-3 proteins in HL-60 and HL-60 R cells determined by Western blot analysis. * p < 0.05. Cell experiments were performed three times independently. [file 12967_2022_3456_MOESM9_ESM.jpg]

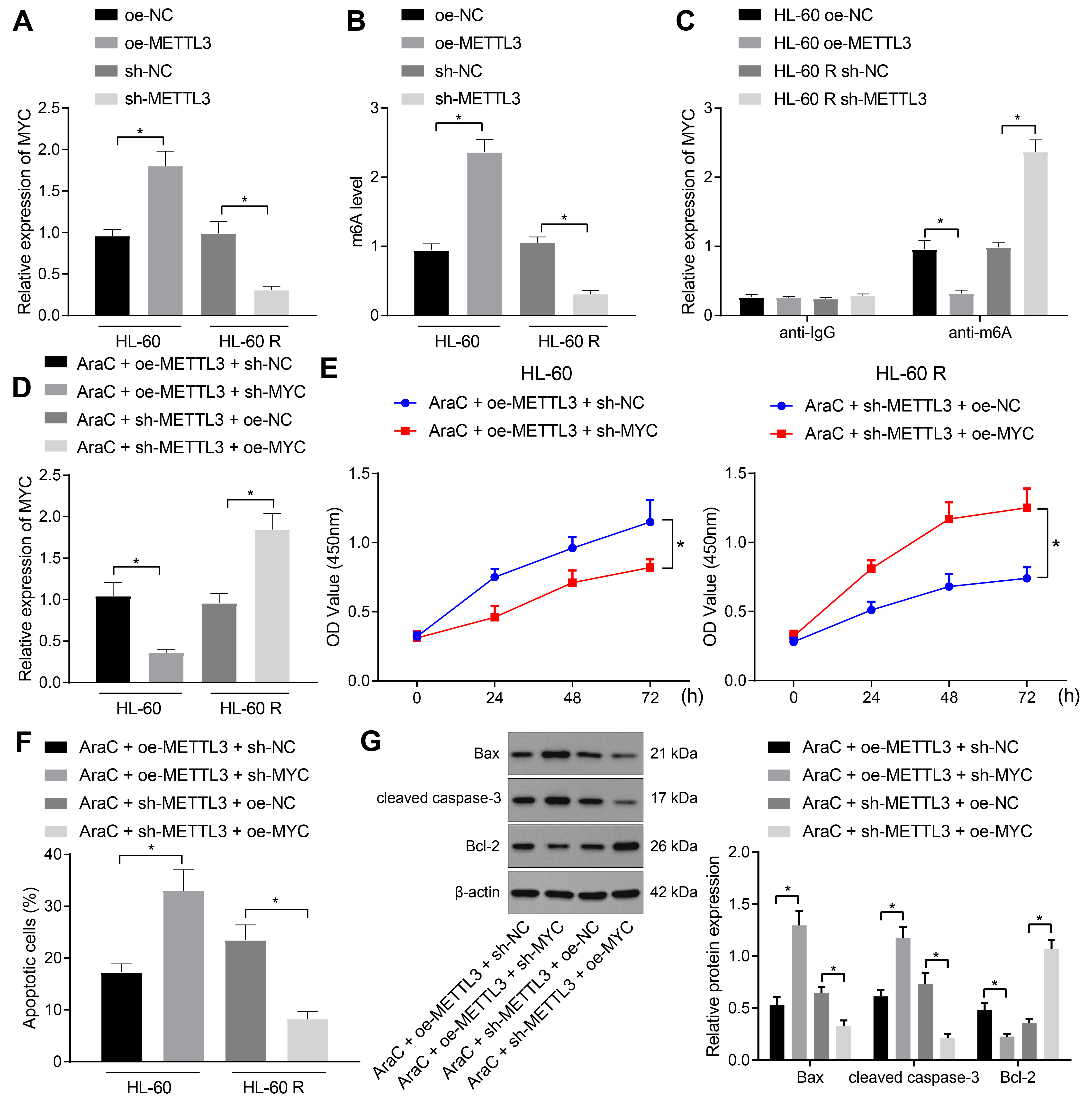

Supplement: Supplementary file 10 — Additional file 10: Figure S5 METTL3 upregulates MYC expression through MYC m6A methylation and reduces the sensitivity of HL-60 cells to AraC. HL-60 cells were transfected with oe-METTL3 and HL-60 R cells were transfected with sh-METTL3. A MYC expression in HL-60 and HL-60 R cells measured by RT-qPCR. B The m6A modification level in Molm13 and Molm13 R cells with methylene blue staining as control detected by Dot blot assay. C Me-RIP and RT-qPCR quantification of the MYC-modified m6A level. HL-60 cells were treated with AraC (2 μM) + oe-METTL3 + sh-MYC, and HL-60 R cells were treated with AraC (2 μM) + sh-METTL3 + oe-MYC. D MYC expression in HL-60 and HL-60 R cells measured by RT-qPCR. E Viability of HL-60 and HL-60 R cells evaluated by CCK-8 assay. F Apoptosis of HL-60 and HL-60 R cells detected by flow cytometry. G Expression of Bcl-2, Bax, and cleaved caspase-3 proteins in HL-60 and HL-60 R cells determined using Western blot analysis.* p < 0.05. Cell experiments were performed three times independently [file 12967_2022_3456_MOESM10_ESM.jpg]

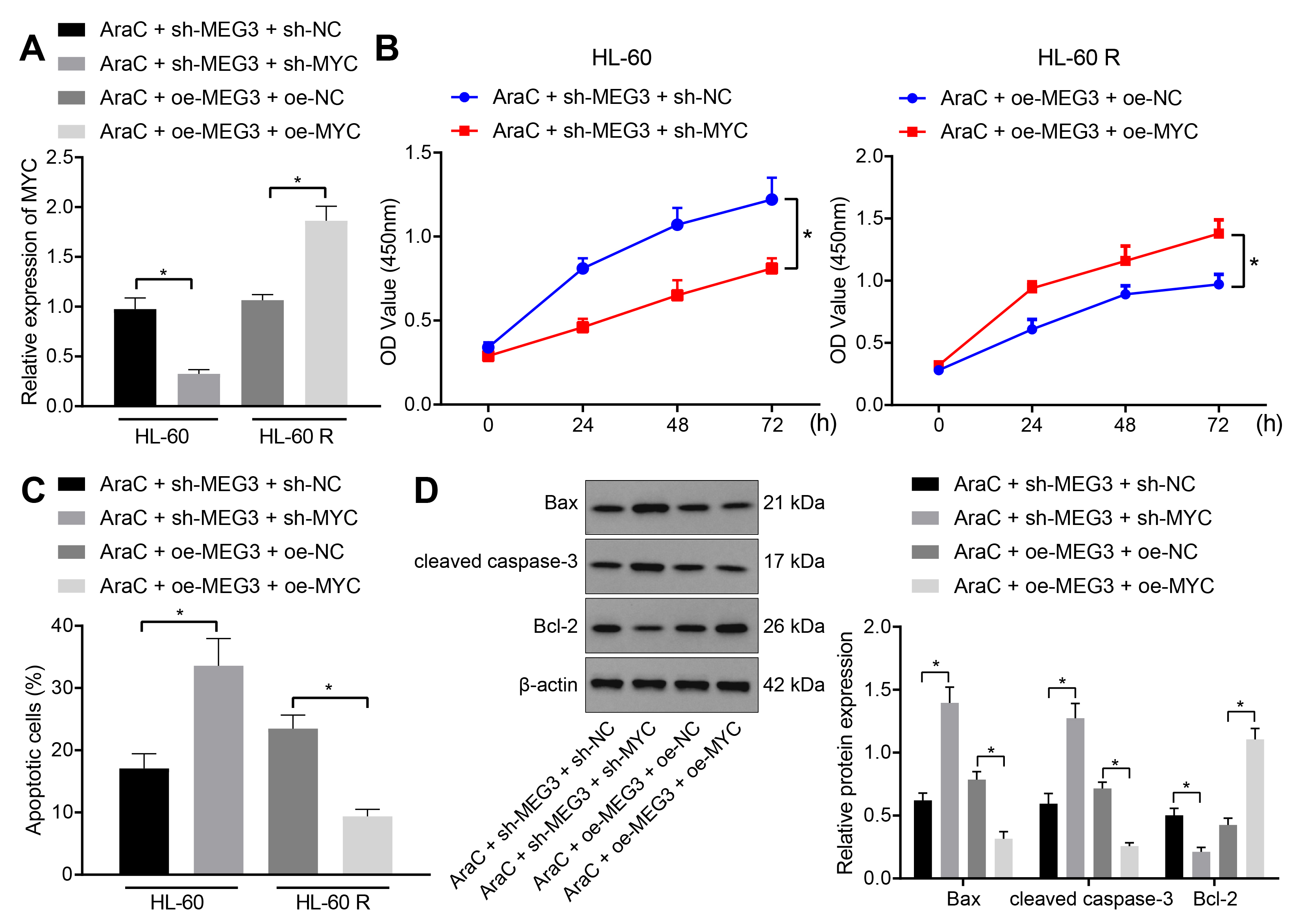

Supplement: Supplementary file 11 — Additional file 11: Figure S6 MEG3 upregulates miR-493-5p and downregulates the METTL3/MYC axis to promote the sensitivity of HL-60 cells to AraC. HL-60 cells were treated with AraC (2 μM) + sh-MEG3 + sh-MYC, and HL-60 R cells were treated with AraC (2 μM) + oe-MEG3 + oe-MYC. A MYC expression in HL-60 and HL-60 R cells was determined by RT-qPCR. B Viability of HL-60 and HL-60 R cells was determined by CCK-8 assay. C Apoptosis of HL-60 and HL-60 R cells was detected by flow cytometry. D The expression of Bcl-2, Bax, and cleaved caspase-3 proteins in HL-60 and HL-60 R cells determined by Western blot analysis. * p < 0.05. Cell experiments were performed three times independently [file 12967_2022_3456_MOESM11_ESM.jpg]

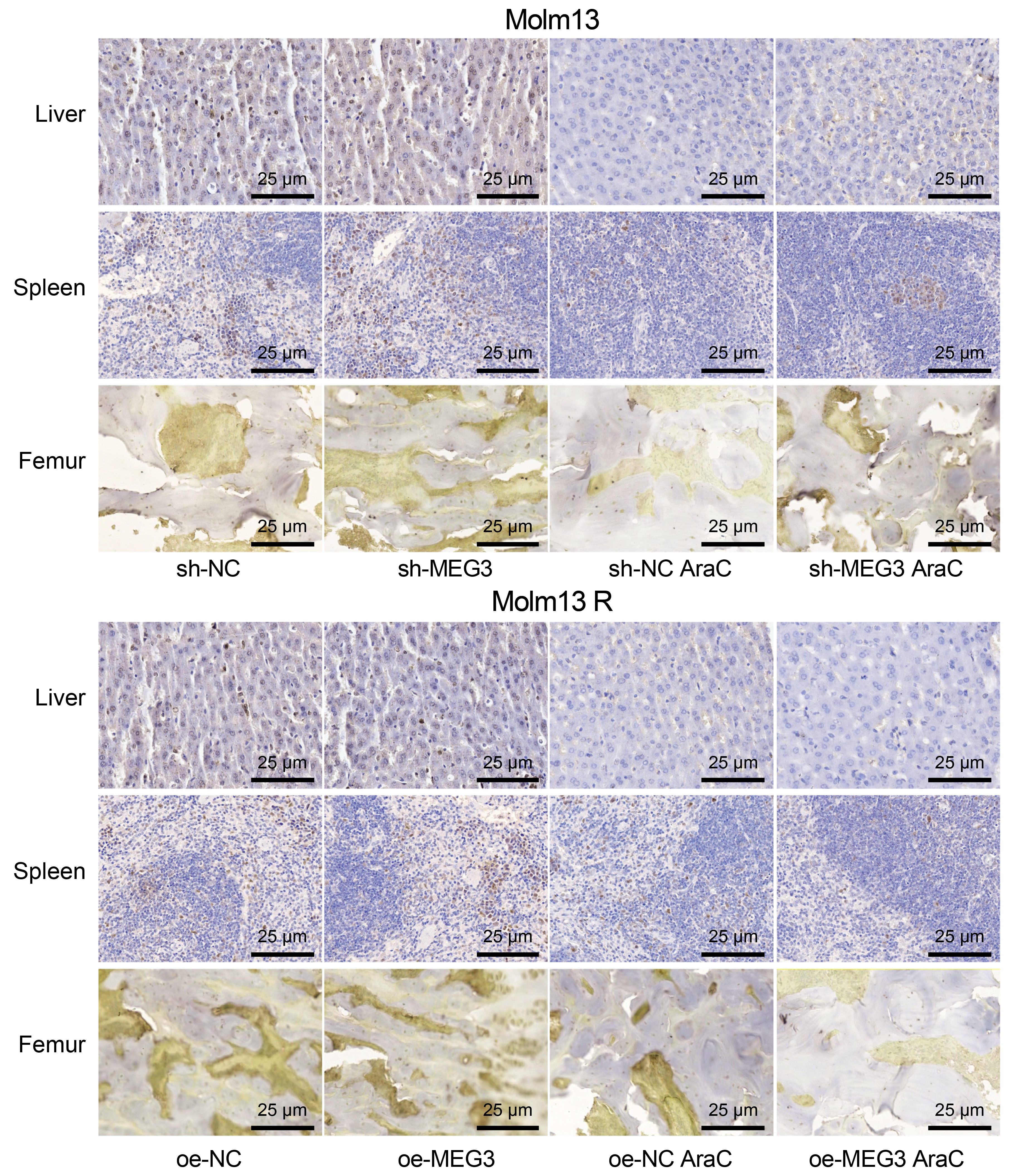

Supplement: Supplementary file 12 — Additional file 12: Figure S7 Representative IHC images of the content of AML cells in liver, spleen, and femur tissues of mice [file 12967_2022_3456_MOESM12_ESM.jpg]
